# Differentiating T2 hyperintensity in neonatal white matter by two-compartment model of diffusional kurtosis imaging

**DOI:** 10.1038/srep24473

**Published:** 2016-04-14

**Authors:** Jie Gao, Xianjun Li, Yanyan Li, Lingxia Zeng, Chao Jin, Qinli Sun, Duan Xu, Bolang Yu, Jian Yang

**Affiliations:** 1 Department of Diagnostic Radiology, The First Affiliated Hospital, Xi’an Jiaotong University, Xi’an 710061, PR China; 2 Department of Biomedical Engineering, School of Life Science and Technology, Xi’an Jiaotong University, Xi’an 710054, PR China; 3 Department of Epidemiology and Health Statistics, School of Public Health, Xi’an Jiaotong University Health Science Center, Xi’an 710061, PR China; 4 Department of MRI Diagnosis, Shannxi Provincial People’s Hospital, Xi’an 710068, PR China; 5 Department of Radiology and Biomedical Imaging, University of California, San Francisco, USA

## Abstract

In conventional neonatal MRI, the T2 hyperintensity (T2h) in cerebral white matter (WM) at term-equivalent age due to immaturity or impairment is still difficult to identify. To clarify such issue, this study used the metrics derived from a two-compartment WM model of diffusional kurtosis imaging (WM-DKI), including intra-axonal, extra-axonal axial and radial diffusivities (D_a_, D_e,//_ and D_e,⊥_), to compare WM differences between the simple T2h and normal control for both preterm and full-term neonates, and between simple T2h and complex T2h with hypoxic-ischemic encephalopathy (HIE). Results indicated that compared with control, the simple T2h showed significantly increased D_e,//_ and D_e,⊥_, but no significant change in D_a_ in multiple premyelination regions, indicative of expanding extra-axonal diffusion microenvironment; while myelinated regions showed no changes. However, compared with simple T2h, the complex T2h with HIE had decreased D_a_, increased D_e,⊥_ in both premyelination and myelinated regions, indicative of both intra- and extra-axonal diffusion alterations. While diffusion tensor imaging (DTI) failed to distinguish simple T2h from complex T2h with HIE. In conclusion, superior to DTI-metrics, WM-DKI metrics showed more specificity for WM microstructural changes to distinguish simple T2h from complex T2h with HIE.

As a higher signal intensity in periventricular and subcortical white matter (WM) than in normal unmyelinated WM on T2 weighted image (T2WI), T2 hyperintensity (T2h) has a high incidence of up to 80% in preterm infants at term-equivalent age (TEA)^1^,^2^, and originally coined as diffuse and excessive high signal intensity (DEHSI). Besides of preterm neonates, it is also a common finding in full-term neonates. Such kind of WM abnormality, particularly combined with multiple parenchymal lesions may affect the following neurodevelopment outcomes^2^. However, the histological and microstructural changes underlying T2h have not yet to be fully elucidated^1^,^2^,^3^,^4^,^5^,^6^. One plausible hypothesis is that T2h represents a prematurity-related developmental phenomenon for its high incidence^1^,^2^ in preterm infants at TEA and disappearance after a postmenstrual age (PMA) of 50 weeks^6^. Conversely, other studies pointed out that it may also represent an early stage of WM injury that has been closely linked with abnormal WM microstructure at term^7^,^8^ and cognitive impairments up to 9 years of age^1^,^9^. From this, it is worthy to clarify the WM microstructure differences between the pure T2h and T2h combined with multiple parenchymal lesions^2^,^6^,^10^,^11^,^12^ (e.g. hypoxic-ischemic encephalopathy, HIE).

Recent advances in magnetic resonance imaging (MRI) have provided more important information about the development in the neonatal WM^13^. Through structural and diffusion tensor imaging (DTI), the myelination process presented three successive stages^14^,^15^: first, fibers organization in fascicles which lead to an increased fractional anisotropy (FA) on DTI but without obvious change on conventional MRI; second, “premyelination” stage by showing a shortening signal intensity on T1 weighted image (T1WI) and a decreased diffusivity, including the mean diffusivity (MD), axial diffusivity (AD) and radial diffusivity (RD), but without significant change in FA due to the oligodendrocytes and membranes proliferation; Third, “true” myelination stage by showing a shortening signal intensity on T2WI, decreased RD and increased FA due to the oligodendrocytes spiral ensheathment around the axon and compact packaged fibers. Since oligodendrocyte precursors (pre-OLs) are particularly vulnerable to a variety of chemical mediators including reactive oxygen species, glutamate, cytokines, and adenosine^16^,^17^,^18^, the myelination process often delays due to pre-OLs loss in the developing brain, especially in the premyelination regions^19^,^20^,^21^,^22^. With respect to axon, a recent histological study further reported that the axon degeneration was restricted to necrotic WM injury rather than non-necrotic WM injury^22^. Thus, it can be concluded that the axon damage may be the critical factor for identifying the severe WM injury.

With respect to the T2h, diffusion weighted imaging (DWI) and DTI have revealed that such WM abnormality is associated with significant region-specific changes, such as decreased FA, and increased apparent diffusion coefficient (ADC), AD and RD^7^,^23^,^24^,^25^. However, it remains the difficulty for DTI in identifying whether these abnormal diffusivities are due to intra- or extra-axonal microstructural changes. As an alternative, diffusional kurtosis imaging (DKI) is a clinically feasible extension of DTI that examines the additional contribution of non-Gaussian diffusion effects as a result of brain microstructural complexity^26^. Recently, a two-compartment non-exchange diffusion model of the WM has been proposed that is suitable for DKI analysis and provides analytical expressions for the intra- and extra-axonal diffusion tensors^27^. Notably, the derived metrics, including intra-axonal diffusivity (D_a_) and extra-axonal axial and radial diffusivities (D_e,//_ and D_e,⊥_), have been demonstrated to improve the understanding of WM alterations in various clinical diseases, such as Alzheimer’s disease^28^,^29^, schizophrenia^30^ and stroke^31^.

Taken together, a key aspect of differentiating the varying T2h may lie in the intra- and extra-axonal microstructure changes of WM. From this, we propose an hypothesis that the simple T2h (i.e. pure T2h) may be due to delayed myelination only, which meant only diffusivity changes in extra-axonal space; being different from simple T2h, the complex T2h (i.e. T2h combined with HIE) may be due to axonal damage which indicates the different microstructural changes (as exhibited in Fig. 1). Therefore, aiming at verifying such hypothesis by using the DKI-WM metrics, the case-control neonate study was conducted to clarify the intra- and extra-axonal microstructure changes between normal control and simple T2h; between simple T2h and complex T2h.

## Results

### Subjects

The study design flowchart is shown in Fig. 2. Of 423 neonates reviewed, 132 neonates with the PMA of 37–42 weeks had received conventional MRI and DKI. Further 13 neonates were excluded from the analysis due to congenital metabolic disorder (*n* = 2), congenital infection (*n* = 3) or motion artifact (*n* = 8). Thus, 92 full-term neonates with gestational age (GA) of 39.18 ± 1.28 weeks and PMA at MRI scan of 40.55 ± 1.45 weeks and 27 preterm neonates with GA of 35.08 ± 2.07 weeks and PMA at MRI scan of 38.58 ± 1.73 weeks were enrolled for MRI interpretation. Following discussion and consultation, 21, 41 and 27 full-term neonates, and 8, 11 and 8 preterm neonates, were allocated respectively to the normal control group, simple T2h group and complex T2h with other abnormality group (detailed gestational ages and PMA at MRI scan in each group were shown on Table 1); 5 full-term neonates with GA of 39.20 ± 1.87 weeks and PMA at MRI scan 40.51 ± 2.01 weeks from complex T2h with other abnormality group were further allocated to the complex T2h with HIE group. The rest 22 full-term neonates and 8 preterm neonates in complex T2h with other abnormality group, and 3 full-term neonates presenting with punctate WM lesions without T2h were excluded from DKI data analysis.

In preterm and full-term neonates, the proportions of T2h were 70.4% (19 of 27) and 73.9% (68 of 92), and the proportions of simple T2h were 40.7% (11 of 27) and 44.6% (41 of 92), respectively. The proportions of T2h and simple T2h did not differ significantly between preterm and full-term neonates (*P* = 0.81, 0.83, respectively). The demographic parameters and clinical details of neonates in control group, simple T2h group and complex T2h with HIE group are shown in Table 1. There were no significant differences in the GA, PMA, birth weight (BW), age at the time of MR scan, presence of small size for GA, gender, or multiple births between the preterm simple T2h and control groups, full-term simple T2h and control groups, or full-term simple T2h and complex T2h with HIE groups. Compared with the full-term simple T2h group, the preterm simple T2h group had significantly lower mean GA, PMA and BW, and a significantly higher age at the time of MR scan.

### Observer agreement in the interpretation of T2h in MRI

The first observer allocated 38, 42 and 36 neonates, respectively, to the control, simple T2h and complex T2h groups, while the second observer allocated 31, 53 and 32 neonates, respectively. On the second review by the first observer, 33, 48 and 35 participants were allocated to the control, simple T2h and complex T2h groups. Thus, the Kappa values for inter- and intra-observer agreements in the classification of MR findings were 0.74 and 0.82, respectively, corresponding to substantial and almost perfect agreement, respectively.

### Tract-based spatial statistics (TBSS) analysis

#### Part I. Differences in WM-DKI metrics between the simple T2h and control groups

**Figure 1:**
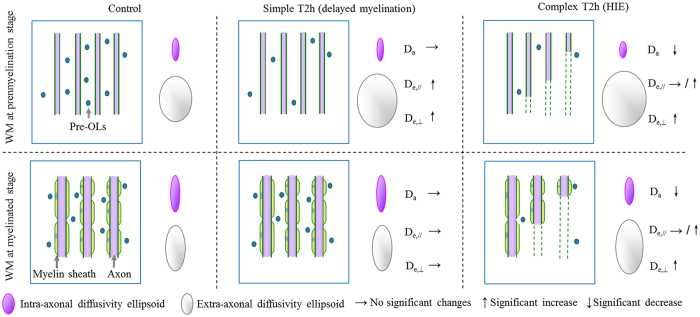
Hypothesis on the relationships between WM-DKI metrics and white mattter (WM) changes of control, simple T2h, complex T2h (combined with HIE) in the premyelination and myelinated stages. Considering their critical roles in WM development and injury, the axons, myelin sheath and oligodendrocyte precursors (pre-OLs) were determined as the main elements of our hypothesis model. As shown in left column, myelination process is mainly depicted by the proliferation of oligodendrocytes lineage precursors and ensheathment of oligodendroglial processes around the axons. As shown in middle column, in case of simple T2h, delayed myelination occurred (presented as the loss of pre-Ols). Thus, compared with control neonates, changes of WM-DKI metrics only presented in extra-axonal spaces rather than intra-axonal spaces at premyelination stage. As shown in right column, in condition of complex T2h with HIE, we hypothesized there existed additional axonal damages, which could lead to the intra-axonal diffusivity decrease in WM at both myelinated and premyelination stages. Beyond, HIE may induce the destructive axons, pre-OLs loss, vasogenic edema and aggregation of microglia and astrocytes; the compound of such changes might lead to the increased extra-axonal radial diffusivity and unchanged or slightly increased extra-axonal diffusivity.

**Figure 2:**
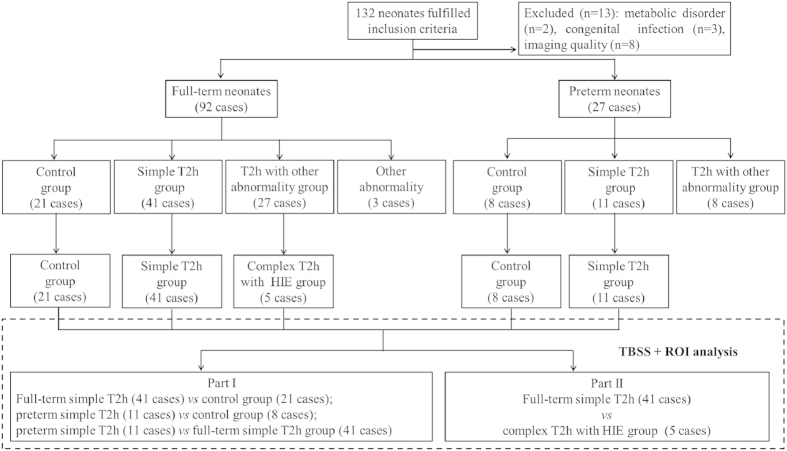
Study design flowchart.

**Figure 3:**
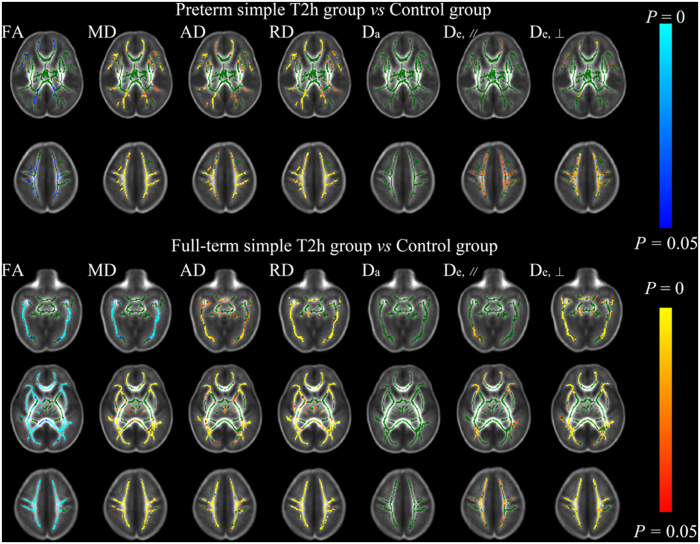
Tract-based spatial statistics for comparisons between the preterm simple T2h and control groups, and the full-term simple T2h and control groups. Regions colored light blue-blue represent significantly decreased voxels (*P* < 0.05) in the simple T2h group compared with the control group, while regions colored yellow-red represent significantly increased voxels (*P* < 0.05) in the simple T2h group compared with the control group. These have been overlaid on the mean FA template with a mean skeleton (green). Compared with the control group, the simple T2h group showed decreased FA and increased MD, AD, RD, D_e,∥_ and D_e,⊥_ in multiple premyelination regions in both preterm and full-term neonates, but no differences were observed in myelinated regions (such as the cerebral peduncle and posterior limb of the internal capsule). Moreover, D_a_ showed no significant change.

As shown in Fig. 3, the preterm simple T2h group showed significantly decreased FA, significantly increased MD, AD, RD, D_e,//_ and D_e,⊥_in the superior corona radiata (SCR) and subcortical deep WM regions of superior frontal gyrus, superior occipital gyrus, precentral gyrus and postcentral gyrus, etc, and no change in D_a_ compared with the control group. In comparisons between the full-term simple T2h and control groups; besides of above regions, similar results were observed in more widespread WM regions, including posterior thalamic radiation (PTR), corpus callosum (CC) and external capsule (EC), etc. However, for both preterm and full-term simple T2h groups compared with corresponding control groups, no differences were observed mainly in the myelinated WM regions at TEA, such as the cerebral peduncle (CP) and posterior limb of the internal capsule (PLIC). The metric changes in the simple T2h group reflect increased diffusivity from the extra-axonal space, which was compatible with delayed myelination (illustrated in Fig. 1). When these diffusional metrics were compared directly between the preterm and full-term simple T2h groups (with GA, PMA and BW as covariates for correction), no differences were found (TBSS analysis not shown), indicating no essential differences between preterm and full-term neonates with the simple T2h.

#### Part II. Differences in WM-DKI metrics between the simple T2h and complex T2h with HIE groups

**Figure 4:**
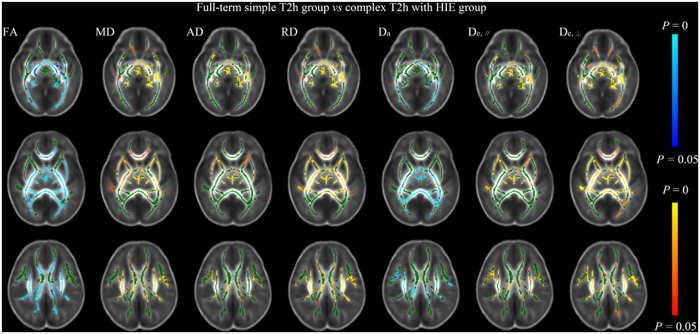
Tract-based spatial statistics for comparisons between full-term simple T2h and complex T2h with HIE groups. Regions colored light blue-blue represent significantly decreased voxels (*P* < 0.05) in the complex T2h with HIE group compared with the full-term simple T2h group, while regions colored yellow-red represent significantly increased voxels (*P* < 0.05). These have been overlaid on the mean FA template with a mean skeleton (green). D_a_ and FA were significantly decreased in both myelinated and premyelination WM regions in the complex T2h with HIE group compared with the full-term simple T2h group, and MD, RD and D_e,⊥_ were increased to differing extents.

Compared with the full-term simple T2h group, the complex T2h with HIE group exhibited significantly decreased D_a_ and FA in the CP, PLIC, PTR, SCR, CC and EC, with mainly increases in MD, RD, and D_e,⊥_ (Fig. 4). The involvement of the CP and PLIC in these changes indicates the extensive axonal damage occurring in both myelinated and premyeliantion WMs in the neonates of HIE, which were different changes from the simple T2h (illustrated in Fig. 1).

### ROI analysis

The PLIC, SCR, CC and EC, which respectively represented projection fibers, commissural fibers and association fibers respectively, were selected as target ROI regions. The results of the ROI analysis (Tables 2, 3, 4, 5) were almost entirely consistent with those of the TBSS analysis. D_a_ was identified as distinguishing between the simple T2h and complex T2h with HIE groups in regions that represented myelinated (the PLIC) and premyelination fibers (the SCR). In addition, all parameters for the PLIC showed no significant differences between the simple T2h and control groups in both preterm and full-term neonates.

## Discussion

In this study, an advanced technique employing a two-compartment WM-DKI model was used to explore the diffusion variation underlying T2h in the neonatal WM. The results provide the novel insights into the possible abnormal changes underlying simple T2h, which were similar between preterm and full-term neonates. Furthermore, above findings of WM-DKI metrics suggest that simple T2h and complex T2h with HIE may originate from differing WM microstructural changes.

With respect to simple T2h, previous DTI studies^7^,^23^,^24^,^25^ have also observed lower FA and higher MD, AD and RD in neonates. However, due to the non-specificity of DTI measures for the intra- and extra-axonal diffusion, it is still difficult to determine what structure alters on earth. Our results regarding the simple T2h group observed the changes of DKI-WM metrics mainly in multiple premyelination regions hinting that the elevated diffusivity was from the extra-axonal space rather than the intra-axonal space; besides, such abnormality was absent in highly anisotropic regions (such as the PLIC and CP, etc ) where show almost complete myelination at the TEA^32^. A more recent DTI study^25^ about T2h (DEHSI) also observed similar patterns of spatial distribution. Specifically, higher diffusivity values and lower FA were found in centrum semiovale and OR, while CP and CST showed no difference between those with or without DEHSI at TEA. It is well known that the normal premyelination process is closely linked to a decrease in brain water content and the proliferation of oligodendrocyte lineage precursors, which showed an overall decrease in diffusivity and increase in FA with age^14^. Hence, the extra-axonal diffusional changes in the opposite way (increase in extra-axonal diffusivity and decrease in FA ) observed in simple T2h were just compatible with the delayed myelination, which might result from a sparsity of oligodendrocytes and a relatively elevated free water content in the extracellular matrix (as illustrated Fig. 1). This speculation was also supported by an animal experiment^22^, in which they found non-necrotic WM injury mainly led to myelination failure but without axonal degeneration. Additionally, we found no obvious differences in the microstructural changes of preterm and full-term neonates with simple T2h, suggesting that these two populations possess similar microstructural alterations.

Targeting the complex T2h with HIE, lower FA and slightly higher MD and RD were found than those with simple T2h. Although these, it was not sufficiently specific to differentiate these two groups since similar diffusivity changes were observed between simple T2h and controls. Notably, being different from simple T2h, D_a_ was markedly decreased in both myelinated and premyelination WM regions in complex T2h with HIE. These observations may hint an extensive breakdown of axons due to these severe WM damage, which was also demonstrated by previous histological studies^22^,^33^. All these may suggest D_a_ to be a specific biomarker for distinguishing these two abnormalities. A similar change in D_a_ has been reported in a study of acute and subacute ischemic lesions^31^, supporting D_a_ as a sensitive and specific biomarker of axonal abnormalities. Furthermore, these findings suggest that the microstructural changes can be more severe in complex T2h, partly explaining the varied neurodevelopmental outcomes observed previously^1^,^2^,^3^,^4^,^5^,^6^.

There were some limitations to this study. First, there is unavoidable subjectivity in the screening of T2h in T2WIs, as reported previously^11^. Although ADC and T2 values have been proposed to improve the accuracy of diagnosing T2h^23^,^24^,^34^, we did not use these approaches due to the lack of a definite and unified standard. However, the inter- and intra-observer agreement in this study (Kappa values 0.74 and 0.82) were receivable in repeatability test. Second, the sample size for neonates with HIE was small due to the low incidence of this injury type at our institution. Moreover, the number of preterm neonates with scans at TEA was also low due to parental concerns. Therefore the inclusion criteria for PMA can be easily met in full-term neonates, the high incidence of T2h in this population may reflect selection bias rather than the real situation. Third, the absence of follow-up for our enrolled neonates necessitates to further validation of our hypothesis.

In conclusion, through using two-compartment WM-DKI metrics, this study provided series of interesting findings about the underlying microstructural changes of T2h: (1) for both preterm and full-term neonates with simple T2h, increased extra-axonal diffusivity and unchanged intra-axonal diffusivity in multiple premyelination WM regions may hint the delayed myelination; (2) while, T2h with HIE showed markedly decreased intra-axonal diffusivity which may be related to axonal damage; (3) superior than conventional DTI metrics, WM-DKI metrics are more specific for identifying the WM microstructural changes (e.g. intra- and extra-axonal diffusivity) in developmental brain.

## Materials and Methods

This single-center retrospective study was approved by the Ethics Committee of the First Affiliated Hospital of Xi’an Jiaotong University, and the written parental consent was obtained prior to scanning. This study was conducted in accordance with the Declaration of Helsinki.

### Subjects

All neonates were consecutively collected from our institution’s neonatal intensive care unit, from December 2010 to February 2014. The neonates that had received conventional MR imaging and DKI at the PMA of 37–42 weeks were included. Any neonates who diagnosed as congenital metabolic disorder, malformation or infection, or had poor MR image quality were excluded from this study. Demographic parameters and clinical details of all the neonates were reviewed and recorded by an experienced neonatal radiologist (Q.L.S. with 10 years of related experience ).

### MR data acquisition

All MR images were obtained using a 3.0 Tesla scanner (Signa HDxt, General Electric Medical System, Milwaukee, WI, USA) equipped with an 8-channel phase array radio-frequency head coil. Chloral hydrate (50 mg/kg) was administered orally for sedation in 30 minutes before the scan. The neonate was kept warm with swaddle and the head immobilized by molded foam. Micro-earplugs and earmuffs were used to protect the hearing. During imaging, the neonate was monitored by the attending pediatrician, and vital signs were monitored continuously.

Three-dimensional fast spoiled gradient-recalled echo T1WIs (repetition time /echo time, 10/4.6 ms) and fast spin-echo T2WIs (repetition time/echo time, 4200/116.4–118.9 ms) were obtained. DKI was acquired using the following parameters: 18 directions; b value = 0, 500, 1000 and 2000 s/mm^2^; repetition time/echo time= 8000/100.2–117.7 ms; slice thickness of 4 mm; field of view, 180 × 180 mm^2^; matrix, 256 × 256; and acquisition time of 8 minutes 42 seconds. The total scan time was less than 30 minutes.

### MR interpretation

MR abnormalities (i.e. T2h and other abnormalities) were screened according to the clinical MR standards^2^. Wherein, T2h was defined as visually higher signal intensity in periventricular and/or subcortical WM than in normal unmyelinated WM on T2WIs; while the “anterior caps” and “posterior arrowheads” would be excluded due to its common high signal intensity^12^. The other abnormalities included cystic encephalomalacia, punctate lesions, a loss of volume of the periventricular WM and corpus callosum, the change of gray matter signal intensity, the widening of the subarachnoid space and intraventricular hemorrhage.

Neonates without any MR abnormalities and no evidences of any clinical episodes that might cause cerebral damage or delayed maturation were allocated to the control group. Neonates with T2h but no above abnormalities were allocated to the simple T2h group. Meanwhile, neonates with both T2h and above abnormalities were allocated to the complex T2h group. Furthermore, in the complex T2h group, the full-term neonates fulfilled both MRI^35^ and clinical diagnostic criteria for HIE^36^ would be allocated to the complex T2h with HIE group. Specifically, the MR diagnostic criteria refers to the focal or diffuse abnormal signal intensities in the bilateral globus pallidus, putamen or thalamus on conventional MRI^35^. And the clinical diagnostic criteria for HIE^36^ includes: A pH ≤ 7.0 or a base deficit ≥ 16 mmol/L on umbilical cord blood or any postnatal blood sample within 1 hour of age; or history of an acute perinatal event and either no blood gas available, or a pH from 7.01 to 7.15 or a base deficit from 10 to 15.9 mmol/L, with a 10-minute Apgar score ≤ 5, or assisted ventilation initiated at birth and continued for at least 10 minutes. The rest neonates in the complex T2h group, and neonates without T2h but with any other abnormalities were excluded.

Two experienced pediatric radiologists (B.L.Y and J.G., respectively with 35 and 7 years of related experience), blinded to the neonatal/perinatal history, evaluated all MR images independently. They reviewed the published written and visual descriptions of the T2h appearances and agreed on its appearances prior to image analysis. The MR images were anonymized and reviewed on the same workstation, with the same window width and level. One month after the initial review, the MR images were reviewed for a second time by one of the observers (J.G.). Disagreements regarding image findings were resolved by discussion and mutual agreement. In the end, the intra- and inter-observer agreements were evaluated.

### MR data processing

Rigid registration and distortion correction were performed after brain extraction^37^,^38^. Artifact-corrupted DWIs were excluded by using an automated method^39^. Diffusion tensor and kurtosis tensor were estimated by using constrained weighted linear least squares (CWLLS)^40^,^41^, according to the following equation^42^:


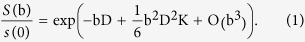


where *S*(b) was the diffusion weighted signal at a particular b value, and *S*(b) the signal without applying any diffusion gradient. *D, K* were the apparent diffusion coefficient and diffusional kurtosis.

Parametric maps of FA, MD, AD, and RD were derived from the diffusion tensor^27^,^43^.

The kurtosis tensor (KT) was transformed by the three eigenvectors of the diffusion tensor (DT)^43^:


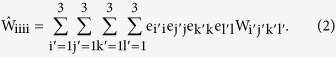


The kurtosis along an individual DT eigenvector can be computed from the transformed KT^43^:


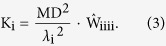


where λ_i_ was the eigenvalues of the DT. RK can be derived by using the eigenvectors on the radial directions^43^.

In the WM model for DKI, let D_a_ and D_e_ represent diffusion tensors in the intra-axonal and extra-axonal spaces. The axonal water fraction (AWF) was denoted by the symbol *f*. The DW signal was described as a function of the b value by the equation^27^:


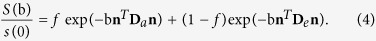


Theoretically, the AWF was calculated based on the maximum kurtosis (K_max_) across all the directions. In practice, it can be estimated by the RK^27^.

The AWF was estimated by the equation^27^:


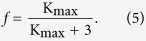


The diffusion coefficients in the intra-axonal and extra-axonal spaces were calculated by the equations^27^:


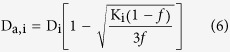



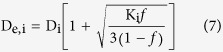


The intra-axonal diffusivity^27^:


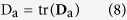


The axial diffusivity in the extra-axonal space^27^:


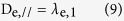


where λ_e,1_ was the primary eigenvalue of D_e_.

The radial diffusivity in the extra-axonal space^27^:


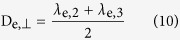


where λ_e,2_, λ_e,3_ were the 2nd, 3rd eigenvalues of D_e_.

Artifacts rejection and tensor estimation were performed by using the in-house software implemented in MATLAB version 7.11.0 (Math Works, Natick, MA, USA).

### TBSS and Region of interest (ROI) analysis

TBSS^44^ was performed by using the optimized pipeline for neonates^45^. All the FA images were normalized to the neonatal Johns Hopkins template^46^. The aligned FA image of each subject was projected onto the mean FA skeleton (threshold = 0.15). Inter-group comparisons of the above metrics were tested with adding covariates (including GA, PMA and BW) in the general linear model. The number of permutations was set at 5000. All tests were taken to be significant at *P* < 0.05 after family-wise error rate (FWE) correction with threshold-free cluster enhancement (TFCE). ROI analysis was also performed based on the Johns Hopkins University WM label atlas^46^. The PLIC, SCR, CC and EC, which respectively represented projection fibers, commissural fibers and association fibers respectively, were selected as target regions.

### Statistical analysis

Statistical analysis was conducted using SPSS for Windows version 17.0 (SPSS, Chicago, IL, USA). Measurement data are reported as means ± standard deviations or medians with ranges, and categorical data as frequencies and percentages. Student’s t-test, one-way analysis of variance, the rank-sum test, Kruskal-Wallis H-test or χ^2^ test, as appropriate, were used for comparisons of demographic data among groups. All parameters from ROI measurements were compared using a general linear model with GA, PMA and BW as covariates. Observer agreement was evaluated by Kappa analysis, and assessment criteria were as follows: 0.00, poor; 0.01–0.20, slight; 0.21–0.40, fair; 0.41–0.6, moderate; 0.61–0.8, substantial; and 0.81–1.0, almost perfect^47^. All statistical tests were two-tailed, and the level of significance was set at 0.05.

## Additional Information

**How to cite this article**: Gao, J. *et al.* Differentiating T2 hyperintensity in neonatal white matter by two-compartment model of diffusional kurtosis imaging. *Sci. Rep.* **6**, 24473; doi: 10.1038/srep24473 (2016).

## Figures and Tables

**Table 1:** Participant demographics.

	*n*	Gestational age (week)#	Postmenstrual age at MR scan (week)#	Birth weight (g)#	Age at MR scan‡(day)	No. with SGA†	No. of male infants†	No. of singletons†
Preterm neonates								
Control group	8	35.87 ± 1.99	38.85 ± 2.31	2490 ± 505	9 (4–97)	1 (12.5)	4 (50.0)	7 (87.5)
Simple T2h group	11	35.21 ± 1.66	38.47 ± 1.39	2150 ± 469	12 (7–65)	0 (0.0)	8 (72.7)	10 (90.9)
Full-term neonates								
Control group	21	39.75 ± 1.02	41.08 ± 1.36	3243 ± 545	7 (3–24)	2 (9.5)	10 (47.6)	21 (100.0)
Simple T2h group	41	38.96 ± 1.45	40.44 ± 1.52	3136 ± 530	8 (1–35)	5 (12.2)	28 (68.3)	37 (90.2)
Complex T2h with HIE group	5	39.20 ± 1.87	40.51 ± 2.01	3236 ± 498	11 (5–16)	0 (0.0)	5 (100.0)	4 (80.00)
*P* value								
Preterm simple T2h *vs.* control group		0.437	0.655	0.149	0.263	0.421	0.377	1.000
Full-term simple T2h *vs.* control group		0.085	0.266	0.734	0.924	1.000	0.114	0.290
Preterm simple T2h *vs.* full- term simple T2h group		<0.001	<0.001	<0.001	0.006	0.571	1.000	1.000
Full-term simple T2h *vs.* complex T2h with HIE group		0.927	0.995	0.917	0.739	1.000	0.301	0.453

Note: SGA, small size for gestational age.

^Note: #^Data presented as means ± SDs;

^‡^data presented as medians and ranges;

^†^data presented as frequencies and percentages.

**Table 2:** Comparisons of DKI-WM metrics of the posterior limb of the internal capsule among study groups.

	FA	MD (×10^−3^ mm/s^2^)	AD (×10^−3^ mm/s^2^)	RD (×10^−3^ mm/s^2^)	D_a_(×10^−3^ mm/s^2^)	D_e,//_ (×10^−3^ mm/s^2^)	D_e,⊥_ (×10^−3^ mm/s^2^)
Preterm neonates							
** **Control group	0.38 ± 0.02	1.16 ± 0.07	1.68 ± 0.08	0.90 ± 0.06	1.06 ± 0.17	1.93 ± 0.12	1.09 ± 0.11
** **Simple T2h group	0.37 ± 0.03	1.18 ± 0.07	1.70 ± 0.07	0.92 ± 0.08	1.21 ± 0.19	1.95 ± 0.10	1.08 ± 0.09
Full-term neonates							
** **Control group	0.39 ± 0.02	1.14 ± 0.05	1.67 ± 0.05	0.87 ± 0.05	1.04 ± 0.09	1.93 ± 0.07	1.07 ± 0.07
** **Simple T2h group	0.38 ± 0.03	1.16 ± 0.05	1.68 ± 0.05	0.90 ± 0.06	1.07 ± 0.17	1.93 ± 0.08	1.08 ± 0.09
** **Complex T2h with HIE group	0.33 ± 0.04	1.21 ± 0.06	1.68 ± 0.08	0.97 ± 0.07	0.87 ± 0.09	1.94 ± 0.08	1.19 ± 0.07
*P* value							
** **Preterm simple T2h *vs.* control group	0.403	0.344	0.415	0.331	0.322	0.380	0.404
** **Full-term simple T2h *vs.* control group	0.095	0.350	0.918	0.215	0.551	0.815	0.483
** **Preterm simple T2h *vs.* full- term simple T2h group	0.564	0.500	0.637	0.474	0.076	0.328	0.896
** **Full-term simple T2h *vs.* complex T2h with HIE group	0.001	0.030	0.781	0.005	0.013	0.622	0.009

Note: data presented as means ± SDs. AD, axial diffusivity; D_a_, intra-axonal diffusivity; D_e,//_, extra-axonal axial diffusivity; D_e,⊥_, extra-axonal radial diffusivity; FA, fractional anisotropy; MD, mean diffusivity; RD, radial diffusivity.

**Table 3:** Comparisons of DKI-WM metrics of the superior corona radiata among study groups

	FA	MD (×10^−3^ mm/s^2^)	AD (×10^−3^ mm/s^2^)	RD (×10^−3^ mm/s^2^)	D_a_ (×10^−3^ mm/s^2^)	D_e,//_ (×10^−3^ mm/s^2^)	D_e,⊥_ (×10^−3^ mm/s^2^)
Preterm neonates							
** **Control group	0.22 ± 0.02	1.41 ± 0.09	1.75 ± 0.09	1.24 ± 0.09	1.21 ± 0.22	1.95 ± 0.09	1.40 ± 0.09
** **Simple T2h group	0.20 ± 0.03	1.58 ± 0.11	1.92 ± 0.10	1.41 ± 0.12	1.47 ± 0.30	2.10 ± 0.10	1.53 ± 0.12
Full-term neonates							
** **Control group	0.25 ± 0.02	1.35 ± 0.07	1.72 ± 0.07	1.16 ± 0.07	1.18 ± 0.13	1.91 ± 0.07	1.34 ± 0.07
** **Simple T2h group	0.22 ± 0.03	1.46 ± 0.09	1.80 ± 0.08	1.28 ± 0.10	1.31 ± 0.24	1.98 ± 0.07	1.45 ± 0.10
** **Complex T2h with HIE group	0.20 ± 0.03	1.51 ± 0.08	1.83 ± 0.08	1.35 ± 0.09	1.03 ± 0.06	2.03 ± 0.07	1.53 ± 0.09
*P* value							
** **Preterm simple T2h *vs.* control group	0.201	0.016	0.011	0.024	0.174	0.009	0.047
** **Full-term simple T2h *vs.* control group	<0.001	<0.001	0.001	<0.001	0.076	0.002	<0.001
** **Preterm simple T2h *vs.* full- term simple T2h group	0.438	0.203	0.208	0.214	0.540	0.047	0.680
** **Full-term simple T2h *vs.* complex T2h with HIE group	0.040	0.132	0.349	0.093	0.011	0.224	0.068

Note: data presented as means ± SDs. AD, axial diffusivity; D_a_, intra-axonal diffusivity; D_e,//_, extra-axonal axial diffusivity; D_e,⊥_, extra-axonal radial diffusivity; FA, fractional anisotropy; MD, mean diffusivity; RD, radial diffusivity.

**Table 4:** Comparisons of DKI-WM metrics of the corpus callosum among study groups.

	FA	MD (×10^−3^ mm/s^2^)	AD (×10^−3^ mm/s^2^)	RD (×10^−3^ mm/s^2^)	D_a_ (×10^−3^ mm/s^2^)	D_e,//_ (×10^−3^ mm/s^2^)	D_e,⊥_ (×10^−3^ mm/s^2^)
Preterm neonates							
** **Control group	0.26 ± 0.02	1.71 ± 0.06	2.18 ± 0.06	1.47 ± 0.06	1.33 ± 0.15	2.51 ± 0.11	1.74 ± 0.08
** **Simple T2h group	0.25 ± 0.01	1.75 ± 0.06	2.23 ± 0.07	1.51 ± 0.07	1.48 ± 0.24	2.56 ± 0.13	1.75 ± 0.08
Full-term neonates							
** **Control group	0.28 ± 0.02	1.62 ± 0.06	2.12 ± 0.07	1.38 ± 0.06	1.30 ± 0.11	2.43 ± 0.90	1.64 ± 0.07
** **Simple T2h group	0.26 ± 0.02	1.68 ± 0.07	2.15 ± 0.07	1.44 ± 0.07	1.36 ± 0.17	2.43 ± 0.11	1.68 ± 0.08
** **Complex T2h with HIE group	0.23 ± 0.03	1.75 ± 0.08	2.18 ± 0.08	1.53 ± 0.09	1.19 ± 0.15	2.46 ± 0.08	1.78 ± 0.09
*P* value							
** **Preterm simple T2h *vs.* control group	0.253	0.398	0.153	0.618	0.310	0.122	0.640
** **Full-term simple T2h *vs.* control group	0.001	0.007	0.149	0.002	0.150	0.780	0.041
** **Preterm simple T2h *vs.* full- term simple T2h group	0.402	0.125	0.021	0.304	0.099	0.001	0.062
** **Full-term simple T2h *vs.* complex T2h with HIE group	0.005	0.028	0.388	0.010	0.024	0.643	0.016

Note: data presented as means ± SDs. AD, axial diffusivity; D_a_, intra-axonal diffusivity; D_e,//_, extra-axonal axial diffusivity; D_e,⊥_, extra-axonal radial diffusivity; FA, fractional anisotropy; MD, mean diffusivity; RD, radial diffusivity.

**Table 5:** Comparisons of DKI-WM metrics of the external capsule among study groups.

	FA	MD (×10^−3^mm/s^2^)	AD (×10^−3^mm/s^2^)	RD (×10^−3^mm/s^2^)	D_a_ (×10^−3^mm/s^2^)	D_e,//_ (×10^−3^mm/s^2^)	D_e,⊥_ (×10^−3^mm/s^2^)
Preterm neonates							
** **Control group	0.21 ± 0.01	1.29 ± 0.08	1.57 ± 0.07	1.15 ± 0.08	1.21 ± 0.20	1.73 ± 0.08	1.27 ± 0.09
** **Simple T2h group	0.20 ± 0.02	1.38 ± 0.08	1.67 ± 0.08	1.24 ± 0.09	1.39 ± 0.25	1.83 ± 0.10	1.35 ± 0.10
Full-term neonates							
** **Control group	0.23 ± 0.01	1.24 ± 0.05	1.54 ± 0.05	1.09 ± 0.05	1.18 ± 0.12	1.70 ± 0.06	1.22 ± 0.05
** **Simple T2h group	0.21 ± 0.02	1.29 ± 0.07	1.57 ± 0.06	1.15 ± 0.08	1.23 ± 0.22	1.73 ± 0.07	1.27 ± 0.09
** **Complex T2h with HIE group	0.19 ± 0.02	1.36 ± 0.05	1.62 ± 0.04	1.23 ± 0.05	1.08 ± 0.09	1.79 ± 0.04	1.37 ± 0.06
*P* value							
** **Preterm simple T2h *vs.* control group	0.343	0.041	0.028	0.053	0.293	0.047	0.074
** **Full-term simple T2h *vs.* control group	0.007	0.036	0.137	0.021	0.495	0.295	0.061
** **Preterm simple T2h *vs.* full- term simple T2h group	0.714	0.296	0.112	0.430	0.122	0.133	0.741
** **Full-term simple T2h *vs.* complex T2h with HIE group	0.004	0.007	0.022	0.005	0.104	0.030	0.005

Note: data presented as means ± SDs. AD, axial diffusivity; D_a_, intra-axonal diffusivity; D_e,//_, extra-axonal axial diffusivity; D_e,⊥_, extra-axonal radial diffusivity; FA, fractional anisotropy; MD, mean diffusivity; RD, radial diffusivity.

## References

[b1] Dyet L. E. *et al.* Natural history of brain lesions in extremely preterm infants studied with serial magnetic resonance imaging from birth and neurodevelopmental assessment. Pediatrics 118, 536–48 (2006). 16882805 10.1542/peds.2005-1866

[b2] Jeon T. Y. *et al.* Neurodevelopmental Outcomes in Preterm Infants: Comparison of Infants with and without Diffuse Excessive High Signal Intensity on MR Images at Near–term-equivalent Age. Radiology 263, 518–26 (2012). 22403166 10.1148/radiol.12111615

[b3] Hart A. *et al.* Neuro-developmental outcome at 18 months in premature infants with diffuse excessive high signal intensity on MR imaging of the brain. Pediatr Radiol 41, 1284–92 (2011). 21681616 10.1007/s00247-011-2155-7

[b4] Kidokoro H., Anderson P., Doyle L., Neil J. & Inder T. High signal intensity on T2-weighted MR imaging at term-equivalent age in preterm infants does not predict 2-year neurodevelopmental outcomes. AJNR Am J Neuroradiol 32, 2005–10 (2011). 21960493 10.3174/ajnr.A2703 PMC7964405

[b5] Parikh N. A. *et al.* Automatically quantified diffuse excessive high signal intensity on MRI predicts cognitive development in preterm infants. Pediatr Neurol 49, 424–30 (2013). 24138952 10.1016/j.pediatrneurol.2013.08.026 PMC3957176

[b6] de Bruïne F. T. *et al.* Clinical implications of MR imaging findings in the white matter in very preterm infants: a 2-year follow-up study. Radiology 261, 899–906 (2011). 22031710 10.1148/radiol.11110797

[b7] Counsell S. J. *et al.* Axial and radial diffusivity in preterm infants who have diffuse white matter changes on magnetic resonance imaging at term-equivalent age. Pediatrics 117, 376–86 (2006). 16452356 10.1542/peds.2005-0820

[b8] Cheong J. L. *et al.* Abnormal white matter signal on MR imaging is related to abnormal tissue microstructure. AJNR Am J Neuroradiol 30, 623–8 (2009). 19131414 10.3174/ajnr.A1399 PMC7051465

[b9] Iwata S. *et al.* Qualitative Brain MRI at Term and Cognitive Outcomes at 9 Years After Very Preterm Birth. Pediatrics 129, E1138–E47 (2012). 22529280 10.1542/peds.2011-1735

[b10] Skiöld B. *et al.* White matter changes in extremely preterm infants, a population-based diffusion tensor imaging study. Acta Paediatr 99, 842–9 (2010). 20132144 10.1111/j.1651-2227.2009.01634.x

[b11] Hart A. R., Smith M. F., Rigby A. S., Wallis L. I. & Whitby E. H. Appearances of diffuse excessive high signal intensity (DEHSI) on MR imaging following preterm birth. Pediatr Radiol 40, 1390–6 (2010). 20333509 10.1007/s00247-010-1633-7

[b12] Maalouf E. F. *et al.* Magnetic resonance imaging of the brain in a cohort of extremely preterm infants. J Pediatr 135, 351–7 (1999). 10484802 10.1016/s0022-3476(99)70133-2

[b13] Ment L. R., Hirtz D. & Huppi P. S. Imaging biomarkers of outcome in the developing preterm brain. Lancet Neurol 8, 1042–55 (2009). 19800293 10.1016/S1474-4422(09)70257-1

[b14] Dubois J. *et al.* Asynchrony of the early maturation of white matter bundles in healthy infants: quantitative landmarks revealed noninvasively by diffusion tensor imaging. Hum Brain Mapp 29, 14–27 (2008). 17318834 10.1002/hbm.20363 PMC6870818

[b15] Dubois J. *et al.* The early development of brain white matter: a review of imaging studies in fetuses, newborns and infants. Neuroscience 276, 48–71 (2014). 24378955 10.1016/j.neuroscience.2013.12.044

[b16] Hagberg H., Peebles D. & Mallard C. Models of white matter injury: comparison of infectious, hypoxic-ischemic, and excitotoxic insults. Ment Retard Dev Disabil Res Rev 8, 30–8 (2002). 11921384 10.1002/mrdd.10007

[b17] Back S. A. *et al.* Selective vulnerability of late oligodendrocyte progenitors to hypoxia-ischemia. J Neurosci 22, 455–63 (2002). 11784790 10.1523/JNEUROSCI.22-02-00455.2002 PMC6758669

[b18] Yoshioka A., Bacskai B. & Pleasure D. Pathophysiology of oligodendroglial excitotoxicity. J Neurosci Res 46, 427–37 (1996). 8950702 10.1002/(SICI)1097-4547(19961115)46:4<427::AID-JNR4>3.0.CO;2-I

[b19] Wang S. *et al.* Mild hypoxic-ischemic injury in the neonatal rat brain: longitudinal evaluation of white matter using diffusion tensor MR imaging. AJNR Am J Neuroradiol 30, 1907–13 (2009). 19749219 10.3174/ajnr.A1697 PMC7051303

[b20] Back S. A. & Rosenberg P. A. Pathophysiology of glia in perinatal white matter injury. Glia 62, 1790–815 (2014). 24687630 10.1002/glia.22658 PMC4163108

[b21] Riddle A. *et al.* Histopathological correlates of magnetic resonance imaging-defined chronic perinatal white matter injury. Ann Neurol 70, 493–507 (2011). 21796666 10.1002/ana.22501 PMC3170499

[b22] Riddle A. *et al.* Differential susceptibility to axonopathy in necrotic and non-necrotic perinatal white matter injury. Stroke 43, 178–84 (2012). 22076007 10.1161/STROKEAHA.111.632265 PMC3246543

[b23] Counsell S. J. *et al.* Diffusion-weighted imaging of the brain in preterm infants with focal and diffuse white matter abnormality. Pediatrics 112, 1–7 (2003). 12837859 10.1542/peds.112.1.1

[b24] Hagmann C. F. *et al.* T2 at MR imaging is an objective quantitative measure of cerebral white matter signal intensity abnormality in preterm infants at term-equivalent age. Radiology 252, 209–17 (2009). 19561257 10.1148/radiol.2522080589

[b25] Leitner Y. *et al.* Diffuse excessive high signal intensity in low-risk preterm infants at term-equivalent age does not predict outcome at 1 year: a prospective study. Neuroradiology 56, 669–78 (2014). 24823447 10.1007/s00234-014-1373-8

[b26] Jensen J. H. & Helpern J. A. MRI quantification of non-Gaussian water diffusion by kurtosis analysis. NMR Biomed 23, 698–710 (2010). 20632416 10.1002/nbm.1518 PMC2997680

[b27] Fieremans E., Jensen J. H. & Helpern J. A. White matter characterization with diffusional kurtosis imaging. NeuroImage 58, 177–88 (2011). 21699989 10.1016/j.neuroimage.2011.06.006 PMC3136876

[b28] Benitez A. *et al.* White matter tract integrity metrics reflect the vulnerability of late-myelinating tracts in Alzheimer’s disease. Neuroimage Clin 4, 64–71 (2014). 24319654 10.1016/j.nicl.2013.11.001 PMC3853114

[b29] Fieremans E. *et al.* Novel white matter tract integrity metrics sensitive to Alzheimer disease progression. AJNR Am J Neuroradiol 34, 2105–12 (2013). 23764722 10.3174/ajnr.A3553 PMC3962262

[b30] Lazar M., Malaspina D., Miles L., Golestani A. M. & Peccerelli N. Altered white matter myelination in chronic schizophrenia, in Annual Meeting of the International Society for Magnetic Resonance in Medicine. (Salt Lake City, Utah, 2013).

[b31] Hui E. S. *et al.* Stroke assessment with diffusional kurtosis imaging. Stroke 43, 2968–73 (2012). 22933581 10.1161/STROKEAHA.112.657742 PMC3479373

[b32] Barkovich A. J. Magnetic resonance techniques in the assessment of myelin and myelination. J Inherit Metab Dis 28, 311–43 (2005). 15868466 10.1007/s10545-005-5952-z

[b33] Wang S. *et al.* Characterization of White Matter Injury in a Hypoxic-Ischemic Neonatal Rat Model by Diffusion Tensor MRI. Stroke 39, 2348–53 (2008). 18535275 10.1161/STROKEAHA.107.509927

[b34] He L. & Parikh N. A. Automated detection of white matter signal abnormality using T2 relaxometry: application to brain segmentation on term MRI in very preterm infants. NeuroImage 64, 328–40 (2013). 22974556 10.1016/j.neuroimage.2012.08.081 PMC3544934

[b35] Okereafor A. *et al.* Patterns of brain injury in neonates exposed to perinatal sentinel events. Pediatrics 121, 906–14 (2008). 18450893 10.1542/peds.2007-0770

[b36] Shankaran S. *et al.* Whole-body hypothermia for neonates with hypoxic-ischemic encephalopathy. N Engl J Med 353, 1574–84 (2005). 16221780 10.1056/NEJMcps050929

[b37] Smith S. M. *et al.* Advances in functional and structural MR image analysis and implementation as FSL. NeuroImage 23, S208–S19 (2004). 15501092 10.1016/j.neuroimage.2004.07.051

[b38] Woods R. P., Grafton S. T., Holmes C. J., Cherry S. R. & Mazziotta J. C. Automated image registration: I. General methods and intrasubject, intramodality validation. J Comput Assist Tomogr 22, 139–52 (1998). 9448779 10.1097/00004728-199801000-00027

[b39] Li X. *et al.* A robust post-processing workflow for datasets with motion artifacts in diffusion kurtosis imaging. PloS one 9, e94592 (2014). 24727862 10.1371/journal.pone.0094592 PMC3984238

[b40] Tabesh A., Jensen J. H., Ardekani B. A. & Helpern J. A. Estimation of tensors and tensor-derived measures in diffusional kurtosis imaging. Magn Reson Med 65, 823–36 (2011). 21337412 10.1002/mrm.22655 PMC3042509

[b41] Veraart J. *et al.* More accurate estimation of diffusion tensor parameters using diffusion kurtosis imaging. Magn Reson Med 65, 138–45 (2011). 20878760 10.1002/mrm.22603

[b42] Jensen J. H., Helpern J. A., Ramani A., Lu H. & Kaczynski K. Diffusional kurtosis imaging: the quantification of non-gaussian water diffusion by means of magnetic resonance imaging. Magn Reson Med 53, 1432–40 (2005). 15906300 10.1002/mrm.20508

[b43] Hui E. S., Cheung M. M., Qi L. & Wu E. X. Towards better MR characterization of neural tissues using directional diffusion kurtosis analysis. NeuroImage 42, 122–34 (2008). 18524628 10.1016/j.neuroimage.2008.04.237

[b44] Smith S. M. *et al.* Tract-based spatial statistics: Voxelwise analysis of multi-subject diffusion data. NeuroImage 31, 1487–505 (2006). 16624579 10.1016/j.neuroimage.2006.02.024

[b45] Ball G. *et al.* An optimised tract-based spatial statistics protocol for neonates: Applications to prematurity and chronic lung disease. NeuroImage 53, 94–102 (2010). 20510375 10.1016/j.neuroimage.2010.05.055

[b46] Oishi K. *et al.* Multi-contrast human neonatal brain atlas: Application to normal neonate development analysis. NeuroImage 56, 8–20 (2011). 21276861 10.1016/j.neuroimage.2011.01.051 PMC3066278

[b47] Bland J. M. & Altman D. G. Statistical methods for assessing agreement between two methods of clinical measurement. Lancet 1, 307–10 (1986). 2868172

